# Supplementary material for: Incarceration status and cancer mortality: A population-based study
Source: PLoS One. 2022 Sep 16;17(9):e0274703. doi: 10.1371/journal.pone.0274703 (PMC9481043; doi:10.1371/journal.pone.0274703)
Supplement: S1 Table — (PDF) [file pone.0274703.s001.pdf]

Appendix 1: List of cancer ICD codes and grouping by organ system

ICD code	Site Group	Cancer Group
C000-C009	Lip	Head and Neck
C019-C029	Tongue	Head and Neck
C079-C089	Salivary Gland	Head and Neck
C040-C049	Floor of Mouth	Head and Neck
C030-C039, C050-C059, C060-C069	Gum and Other Mouth	Head and Neck
C110-C119	Nasopharynx	Head and Neck
C090-C099	Tonsil	Head and Neck
C100-C109	Oropharynx	Head and Neck
C129, C130-C139	Hypopharynx	Head and Neck
C140, C142, C148	Other Oral Cavity and Pharynx	Head and Neck
C150-C159	Esophagus	Gastrointestinal
C160-C169	Stomach	Gastrointestinal
C170-C179	Small Intestine	Gastrointestinal
C180	Cecum	Gastrointestinal
C181	Appendix	Gastrointestinal
C182	Ascending Colon	Gastrointestinal
C183	Hepatic Flexure	Gastrointestinal
C184	Transverse Colon	Gastrointestinal
C185	Splenic Flexure	Gastrointestinal
C186	Descending Colon	Gastrointestinal
C187	Sigmoid Colon	Gastrointestinal
C188-C189, C260	Large Intestine, NOS	Gastrointestinal
C199	Rectosigmoid Junction	Gastrointestinal
C209	Rectum	Gastrointestinal
C210-C212, C218	Anus, Anal Canal and Anorectum	Gastrointestinal

C220	Liver	Gastrointestinal
C221	Intrahepatic Bile Duct	Gastrointestinal
C239	Gallbladder	Gastrointestinal
C240-C249	Other Biliary	Gastrointestinal
C250-C259	Pancreas	Gastrointestinal
C480	Retroperitoneum	Gastrointestinal
C481-C482	Peritoneum, Omentum and Mesentery	Gastrointestinal
C268-C269, C488	Other Digestive Organs	Gastrointestinal
C300-C301, C310-C319	Nose, Nasal Cavity and Middle Ear	Head and Neck
C320-C329	Larynx	Head and Neck
C340-C349	Lung and Bronchus	Thoracic
C384	Pleura	Thoracic
C339, C381-C383, C388, C390, C398, C399	Trachea, Mediastinum and Other Respiratory Organs	Thoracic
C400-C419	Bones and Joints	Sarcoma
C380, C470-C479, C490-C499	Soft Tissue including Heart	Sarcoma
C440-C449	Melanoma of the Skin	Skin
C440-C449	Other Non-Epithelial Skin	Skin
C500-C509	Breast	Breast
C530-C539	Cervix Uteri	Female Reproductive (gynecology)
C540-C549	Corpus Uteri	Female Reproductive (gynecology)
C559	Uterus, NOS	Female Reproductive (gynecology)
C569	Ovary	Female Reproductive (gynecology)
C529	Vagina	Female Reproductive (gynecology)
C510-C519	Vulva	Female Reproductive (gynecology)
C570-C579, C589	Other Female Genital Organs	Female Reproductive (gynecology)
C619	Prostate	Male Reproductive
C620-C629	Testis	Male Reproductive
C600-C609	Penis	Male Reproductive

C630-C639	Other Male Genital Organs	Male Reproductive
C670-C679	Urinary Bladder	Urinary (non-reproductive)
C649, C659	Kidney and Renal Pelvis	Urinary (non-reproductive)
C669	Ureter	Urinary (non-reproductive)
C680-C689	Other Urinary Organs	Urinary (non-reproductive)
C690-C699	Eye and Orbit	Central Nervous System (CNS)
C710-C719	Brain	Central Nervous System (CNS)
C710-C719	Cranial Nerves Other Nervous System	Central Nervous System (CNS)
C739	Thyroid	Head and Neck
C379, C740-C749, C750-C759	Other Endocrine including Thymus	Head and Neck
C024, C098-C099, C111, C142, C379, C422, C770-C779	Hodgkin - Nodal	Leukemia & Lymphoma
All other sites	Hodgkin - Extranodal	Leukemia & Lymphoma
C024, C098, C099, C111, C142, C379, C422, C770-C779	NHL - Nodal	Leukemia & Lymphoma
All sites except C024, C098-C099, C111, C142, C379, C422, C770-C779	NHL - Extranodal	Leukemia & Lymphoma
	Myeloma	Leukemia & Lymphoma
	Acute Lymphocytic Leukemia	Leukemia & Lymphoma
C420, C421, C424	Chronic Lymphocytic Leukemia	Leukemia & Lymphoma
	Other Lymphocytic Leukemia	Leukemia & Lymphoma
	Acute Myeloid Leukemia	Leukemia & Lymphoma
	Chronic Myeloid Leukemia	Leukemia & Lymphoma
	Other Myeloid/Monocytic Leukemia	Leukemia & Lymphoma
	Acute Monocytic Leukemia	Leukemia & Lymphoma
	Other Acute Leukemia	Leukemia & Lymphoma
	Aleukemic, subleukemic and NOS	Leukemia & Lymphoma
	Mesothelioma	Thoracic
	Kaposi Sarcoma	Sarcoma
	Miscellaneous	Other
