# Supplementary material for: Incarceration status and cancer mortality: A population-based study
Source: PLoS One. 2022 Sep 16;17(9):e0274703. doi: 10.1371/journal.pone.0274703 (PMC9481043; doi:10.1371/journal.pone.0274703)
Supplement: S2 Table — (PDF) [file pone.0274703.s002.pdf]

S2 Table: Length of incarceration and average time to cancer diagnosis

	Status at cancer diagnosis				
	Incarcerated (N=239)		Post-incarceration (N=479)		
	mean	SD	mean	SD	p-value
**Length of incarceration for the target DOC episode (in years)**	4.63	6.14	1.05	1.08	p<0.000
**Time between release and diagnosis (in months)**			5.06	3.47	
**Time between incarceration and diagnosis (in years)**	3.63	5.67			
**Age at first incarceration**	31.35	15.30	33.38	13.87	p=0.074
**Lifetime incarceration before target DOC episode, (in years)**	10.21	8.98	4.8	6.64	p<0.000
**Proportion of life involved with DOC**	0.41	0.22	0.38	0.21	p=0.104
