# Supplementary material for: Incarceration status and cancer mortality: A population-based study
Source: PLoS One. 2022 Sep 16;17(9):e0274703. doi: 10.1371/journal.pone.0274703 (PMC9481043; doi:10.1371/journal.pone.0274703)
Supplement: Attachment — Submitted filename: PLOS-One_CTIncarcerationCancer.docx [file pone.0274703.s003.docx]

**Incarceration status and cancer mortality: A population-based study**

**Peer review**

This study links Connecticut incarceration data to state cancer registry data to determine cancer types and survival rates for individuals who were incarcerated, previously incarcerated in the prior 12 months and never incarcerated at the time of their diagnosis. They find that incarcerated and formerly incarcerated patients were more likely to be diagnosed with distant disease and had worse overall survival than never-incarcerated patients.

Overall, the study has a key strength in using an incarceration data source that appears to include all non-federal jails and prisons in the state. The data linkage likely required an arduous process for approvals and linkage which itself is an accomplishment. This is a thoughtful and technically well-done study. The comments below are suggestions to clarify a few points and draw out some implications from the data that might benefit readers.

Methods

Page 6, line 101: Incarceration data was from 2005-2016. Was this linked to cancer registry data from the same time period?

Page 7, lines 127-131: How were patients categorized if they were previously incarcerated > 1 year from their cancer diagnosis?

Page 7, line 141-142. Screenable cancers: Recognizing the explanation provided in the limitations section, consider adding lung and liver cancer to the screenable cancers. Though these screening mechanisms are not part of incarceration screening guidelines (e.g. NCCHC, federal BOP), inclusion of these cancer types then leads to data that helps illustrate the extent to which the current cancer burden can be ameliorated with all available screening modalities.

Page 9, lines 166-168. Consider using cancer category as a variable in place of screenable cancers as a variable in the overall survival model. Simplifying cancer types to “screening cancers” may mask significant imbalance of cancers like cervical, breast and colon that have very different prognoses. For instance, never-incarcerated has 16% breast cancer vs 2-5% for the other groups and breast cancer generally has a favorable prognosis compared to many other cancers. Adding at least a sensitivity analysis using cancer category in place of screenable cancer can ensure that cancer mix is not explaining survival differences. This variable could also be included in the survival model for the subset of screenable cancers for the same reason (results presented on page 11, line 206)

If available, a breakdown of cancer diagnosis / survival by incarceration in jail vs prison (if those are separate institutions within the state) would be beneficial.

Results

Page 9: Please provide median age of diagnosis.

Page 10, line 190: “For screenable cancers, 58.8% were diagnosed at a late stage…”. Please move the definition of “late stage” up from the next sentence. Consider providing this data for distant disease instead of late disease, so it can be compared to the 42.7% data point in the previous sentence. I personally think of “late stage” as representing incurable, distant disease rather than also encompassing locally advanced disease that is still potentially curable.

Thinking about how this data can be used to direct implementation of interventions to improve cancer care:

- Page 10 line 192-194: Among incarcerated patients with a screenable cancer that were diagnosed with a late stage, the median length of incarceration was 5.9 years, but what percentage of patients had been incarcerated for 1 year or longer? It is another way of illustrating what proportion of cancers might be caught earlier.
- Among incarcerated and formerly incarcerated patients with screenable cancers diagnosed at a late stage, what is the breakdown of cancer type? This determines which screen programs need to be beefed up.

The authors mention Medicaid expansion in the discussion – if the registry reports insurance status at diagnosis for recently incarcerated and the general population, that would be interesting to see.
